# Association between Non-Alcoholic Fatty Liver Disease and Mediterranean Lifestyle: A Systematic Review

**DOI:** 10.3390/nu14010049

**Published:** 2021-12-23

**Authors:** Catalina M Mascaró, Cristina Bouzas, Josep A Tur

**Affiliations:** Research Group on Community Nutrition and Oxidative Stress, University of the Balearic Islands-IUNICS, IDISBA & CIBEROBN (Physiopathology of Obesity and Nutrition CB12/03/30038), 07122 Palma de Mallorca, Spain; c.mascaro@uib.es (C.M.M.); cristina.bouzas@uib.es (C.B.)

**Keywords:** non-alcoholic fatty liver disease, mediterranean diet, physical activity, mediterranean lifestyle, metabolic syndrome

## Abstract

Background and Aims: Non-alcoholic fatty liver disease (NAFLD) is an excessive accumulation of fat in the liver without alcohol abuse. It is linked to metabolic syndrome (MetS) and no pharmacological treatment exists. This systematic review aims to assess evidence about the effect of Mediterranean lifestyle on the prevention and reversion of NAFLD. Methods: A systematic literature search was performed in MEDLINE via Pubmed. MeSH terms used were: non-alcoholic fatty liver disease [MeSH Major Topic] AND metabolic syndrome [MeSH Term] AND (Diet, Mediterranean [MeSH Term]) OR (Exercise [MeSH Term]). (PROSPERO ID: 2021 CRD42021289495). Results: Thirteen articles were selected and divided into two categories (four focused on Mediterranean diet and NAFLD and nine focused on Mediterranean diet, physical activity, and NAFLD). Information of clinical endpoints was based on NAFLD, as well as MetS, body mass index, fasting glycemia, obesity, cholesterol, triglycerides, transaminases, albuminuria, and hepatic steatosis, among others. All studies found beneficial associations between the clinical parameters of NAFLD/MetS and following a Mediterranean diet and regular physical activity. Conclusions: An effective treatment that prevents, and even reverses, NAFLD is to adapt lifestyle to the Mediterranean one, based on a Mediterranean diet and regular physical activity.

## 1. Introduction

Non-alcoholic fatty liver disease (NAFLD) is defined as the accumulation of excessive fat in the liver not linked to alcohol abuse [1]. The main cause of the development of NAFLD is the hepatic accumulation of free fatty acids and triglycerides, which generates oxidative stress, inflammation, and the progression of steatohepatitis and fibrosis in the liver. People with a higher probability of developing this disease are those with type 2 diabetes mellitus, metabolic syndrome (MetS), and obesity (33–50%, 90% and 74%, respectively) [2,3]. Several authors consider NAFLD as being the hepatic expression of MetS [4]. Accordingly, there has recently emerged the concept of metabolic dysfunction-associated fatty liver disease (MAFLD) [5], which is defined as the expression of NAFLD associated with a cluster of metabolic pathologies expressed at the same time. These pathologies may be hypertension, high levels of plasma triglyceride, and high-density lipoprotein (HDL)-cholesterol, as well as type 2 diabetes mellitus, obesity, and altered insulin resistance [6].

A Mediterranean diet has many healthy components: it decreases the risk of cardiovascular disease, MetS, cancer, and overall mortality [7,8]. The benefits of following a Mediterranean diet on NAFLD have also been described because it provides antioxidants and anti-inflammatory nutrients useful in slowing the development of hepatic steatosis [9,10]. A high adherence to a Mediterranean diet improves liver fat content, fibrosis, and insulin sensitivity [11].

Another important aspect of a Mediterranean lifestyle is the practice of regular physical activity. Nowadays, physical inactivity has been recognized as a public health problem, being the fourth-leading risk factor for mortality according to the World Health Organization (WHO) [12]. Maintaining an active lifestyle has many benefits in relation to health. It decreases lipid disorders, such as a high plasma level of triglycerides and low HDL-cholesterol, as well as a decrease in weight and blood pressure [13]. High levels of physical activity decrease type 2 diabetes mellitus, MetS, and NAFLD [14,15], while it is very usual that people with metabolic disorders also engage in low regular physical activity [16,17]. Exercise intensity is also important in reducing MetS risk factors [12]; 3–5 sessions per week of moderate or vigorous physical activity, an equivalent of 150–200 min per week, has been shown to have decreased the development of NAFLD [18].

A Mediterranean lifestyle based on regular physical activity and following a Mediterranean diet is essential to decrease MetS [7,17,19].

Keep in mind how important it is to follow a Mediterranean diet, as well as having regular physical activity. Both have the same relevance, though most studies only delve into Mediterranean diet and mention physical activity, or vice versa, or talk about diet without specifying its type. Therefore, this review delves into both factors.

The aim of the current systematic review was to assess evidence on the effect of Mediterranean lifestyle on the prevention and reversion of NAFLD.

## 2. Methods

The Preferred Reporting Items for Systematic Reviews and Meta-Analyses (PRISMA) [20] guidance was followed, with the protocol of this systematic review registered in the international prospective register of systematic reviews (PROSPERO ID: 2021 CRD42021289495). The systematic review was performed up to October 2021. Searched literature has been retrieved from the MEDLINE database, via PubMed, using the following combination of Medical Subject Headings (MeSH) terms: non-alcoholic fatty liver disease [MeSH Major Topic] AND metabolic syndrome [MeSH Term] AND (Diet, Mediterranean [MeSH Term]) OR (Exercise [MeSH Term]). Two search filters were applied: studies written in English and adult subjects (age >19 years). No further restrictions were applied. The search was standardized and carried out by two independent reviewers who proceeded initially to read the titles, then the summaries, and finally the complete article. In those articles with disagreement, the search was realized in an inverse manner. The search was complemented by reviewing the bibliographic references included in each retrieved article. If any original article was found, it was included in the review.

**Figure 1:**
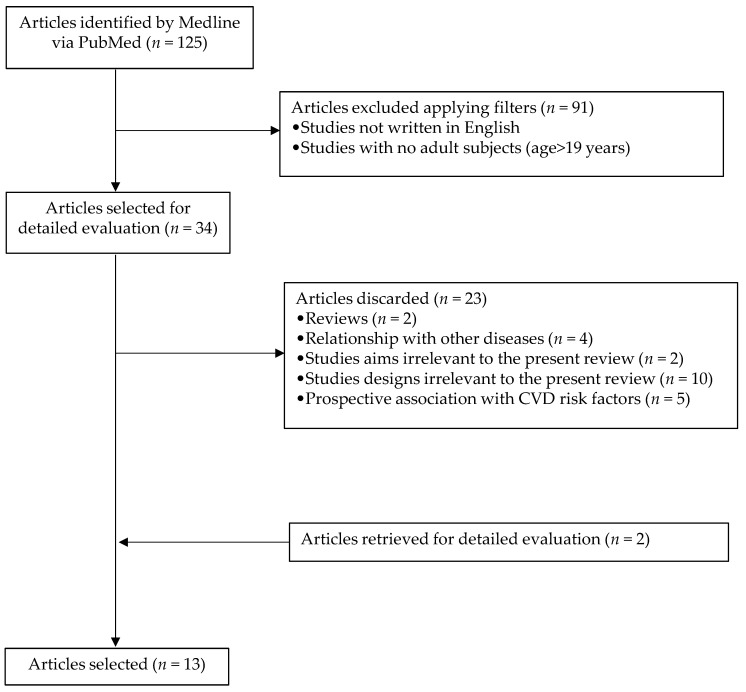
Flowchart highlighting the selection process of articles.

The search offered a total of 125 articles, of which, when applying the above filters, 34 were selected. Then reviews, records related to other diseases, irrelevant study objectives for the current review, irrelevant study designs for this review, and prospective association with cardiovascular disease risk factors were excluded. Two more articles were retrieved after a detailed evaluation of selected papers. Finally, 13 articles were included in the systematic review. The process used to identify and select articles is shown in Figure 1.

The 13 selected studies were finally divided into two categories to facilitate their identification and understanding. Four articles focused on Mediterranean diet and NAFLD, while the remaining nine articles focused on Mediterranean diet, physical activity, and NAFLD.

## 3. Results

Of the four articles that focused on a Mediterranean diet and NAFLD, three were cross-sectional studies, while the fourth was a retrospective case–control study. Two of these were carried out in the Asian area, with a considerable sample size (both exceeded 1000 individuals), while the others were carried out in Europe, with a significantly lower sample size. All studies analyzed the adult population, aged between 34 and 57 years, except one that included subjects older than 18 years.

Articles that focused on a Mediterranean diet, physical activity, and NAFLD showed different designs: three were cross-sectional studies, two were randomized controlled trials, plus there was a cohort study, a controlled clinical study, an observational study, and a prospective observational study. Most took place in Europe, although three were in Asia and one in North America. Sample size was very different between studies; some did not reach 100 individuals, while others showed a sample size greater than 100, and one even exceeded 5000 participants. The studies focused on the adult population, with ages between 35 and 75 years, although one study only included ≥60-year-old subjects and another included ≥26-year-old people.

Most participants included in the reviewed studies were diagnosed with NAFLD, but three studies also included participants without a diagnosis of NAFLD [21,22,23] and five also included participants with MetS [21,24,25,26,27]. Analyses were mainly performed on the study sample as a whole, except for studies in which there were participants without NAFLD. Results were obtained comparing groups with and without NAFLD. The presentation of results was based on hard clinical endpoints of NAFLD, such as fasting glycemia, obesity, adipokines, waist circumference, low-density lipoprotein-cholesterol (LDL) and HDL-cholesterol, triglyceridemia, body mass index, hepatic steatosis index/lipid accumulation, homeostatic model assessment-insulin resistance, transaminases, albuminuria, and hepatic steatosis grade. The results are shown in Table 1.

### 3.1. Results from Studies on Mediterranean Diet and NAFLD

There were four studies that mainly focused on Mediterranean diet and NAFLD [21,23,27,28]. A cross-sectional study [21] compared participants with and without NAFLD. Results showed an increased fasting glycemia and prevalence of MetS in participants with NAFLD. A second cross-sectional study [27] assessed adherence to a Mediterranean diet by MedDiet Score and showed a clear relationship between Mediterranean diet and the improvement of MetS. It showed a positive association between MetS and consumption of red meat and refined grains, while this association was negative between MetS and MedDiet Score and consumption of whole grains. The last cross-sectional study of this section [28] showed the importance of high adherence to a Mediterranean diet as an independent protective factor for liver fibrosis and non-alcoholic steatohepatitis in overweight participants. Finally, a retrospective case–control study [23] could not demonstrate an association between nut consumption, as part of a Mediterranean diet, and NAFLD risk in the overall sample, although there was a significant inverse association between high nut consumption and NAFLD in the highest quartile nut consumption of the men’s sample.

### 3.2. Results from Studies on Mediterranean Diet, Physical Activity, and NAFLD

Nine studies analyzed the association between Mediterranean diet, physical activity, and NAFLD; four of them studied recruited people as a single group [22,25,29,30], two classified participants into two groups [24,31], two analyzed hand-grip strength [32,33], and one assessed renal outcomes [26]. Most studies agreed that a Mediterranean diet and physical activity improved all or some of NAFLD risk factors: weight reduction [22,29,30], body mass index [24,29,30], waist circumference [24,29,30], triglyceridemia [22,29], LDL-cholesterol [29,30,31], impaired fasting glycemia [22,29], visceral adipose index and fatty liver index [29], lipid accumulation [14,29], homeostatic model assessment-insulin resistance [29,30,31]; increased HDL-cholesterol [22,29]; normalization of alanine aminotransferase [22,29,30]; reduction of gamma glutamyl transferase [30]; and improvement of steatosis [24,29,30,31].

In one study, researchers found a reduction of weight; resolution of hypertriglyceridemia, low HDL-cholesterol, and impaired fasting glycemia; and normalization of alanine aminotransferase in men with severe obesity and NAFLD [22]. In another study, adherence to a Mediterranean diet and increased legume consumption were inversely associated with hepatic steatosis index values [25]. In two cases, hepatic steatosis index values decreased in people who engaged in physical activity [24,25]. Only one study pointed out leptin and adiponectin as markers of steatosis reduction [30].

A total reduction of steatosis was shown in nine participants [29], while another study only showed a reduction in the right lobe of the liver [24]. In this last case, changes were observed in participants with a Mediterranean diet, exercise, and a dietary adjunct (two tablespoons per day of a nutraceutical containing 219 mg of Eurosil 85^®^ per tablespoon). When the dietary adjunct was not administered, no changes were observed [24]. Subjects who submitted to 12 weeks of resistance exercise and a Mediterranean diet displayed an increased muscle mass and fat-free mass, and decreased insulin and ferritin levels, while the control group only displayed a reduction of LDL-cholesterol [31].

High hand-grip strength was associated with a decreased risk of NAFLD [33]. Moreover, NAFLD indexes, such as a simple NAFLD score, hepatic steatosis index, NAFLD fibrosis score, and fibrosis 4 calculator, decreased linearly when hand-grip strength increased [32].

Only one article studied the efficacy of lifestyle intervention on the decrease of MetS and NAFLD, but also if these reductions could influence renal outcomes. They divided participants into three groups, each with a specific intervention of diet and physical activity intervention, but no significant differences were identified between these groups. The most significant results were a decreased urinary albumin-to-creatine ratio in participants with increased levels at baseline, but without changes in liver fat; decreased estimated glomerular filtration in participants with hyperfiltration at baseline, associated with low liver fat and insulin resistance and high energy expenditure; energy expenditure decreased hepatic fat accumulation and insulin resistance which is equal to low glomerular hyperfiltration; and a reduction of increased albuminuria without associated reduced liver fat [26].

## 4. Discussion

NAFLD is the accumulation of fat in the liver without alcohol abuse [21]. Nowadays, no definitive therapy has been found to treat NAFLD; the principal treatment is to adopt a Mediterranean lifestyle following a Mediterranean diet and regular physical activity [34]. This helps to reduce NAFLD risk factors, making possible the prevention or regression of this metabolic disease. NAFLD has begun to be known as metabolic dysfunction-associated fatty liver disease or MAFLD because this liver disease is associated with metabolic dysfunctions [6].

### 4.1. Mediterranean Diet and NAFLD

The literature highlights the benefits of a Mediterranean diet on metabolic diseases such as MetS, type 2 diabetes mellitus, and cardiovascular disease, thanks to its components rich in antioxidants, monounsaturated fatty acids, saturated fatty acids, animal protein, and fiber [7]. To understand how a Mediterranean diet can improve MetS, it is necessary to know that MetS is the expression of three or more metabolic disruptions: abdominal obesity, high triglyceridemia, hypertension, high fasting glycemia, and low HDL-cholesterol. So, a Mediterranean diet can improve MetS status because it is able to curtail some or all the disorders associated with MetS [35]. The cluster of these individual alterations also carries a risk for the development of type 2 diabetes mellitus and cardiovascular disease. A Mediterranean diet benefits MetS, type 2 diabetes mellitus, and cardiovascular disease because it improves the parameters that cause these three major pathologies. It decreases hypertension, obesity, and hypercholesterolemia, thus preventing or improving coronary risk. It also decreases lipidemia as well as glycemia, protects against type 2 diabetes mellitus, and then improves MetS. A Mediterranean diet achieves this effect thanks to its anti-inflammatory and antioxidant components, from foods such as olive oil, whole grains, fruits, vegetables, and fish [36].

Polyphenols are another component of a Mediterranean diet that have very positive effects on human health. They are found in foods typical of this diet, such as olive oil, walnuts, and red wine, with the latter always consumed in moderation. Their beneficial effects take place because they act by regulating genes and signaling pathways that intervene on oxidative stress, inflammation, atherosclerosis, and mitochondrial function, keeping in mind that mitochondrial dysfunction is directly related to obesity. Thus, some studies show that the consumption of polyphenols induces an increase of messenger RNA of those genes involved in pathways that regulate obesity, type 2 diabetes mellitus, and dyslipidemia. They have a positive influence on obesity because they reduce the accumulation of intracellular triglycerides and down-regulate the expression of genes linked to adipogenesis and adipocyte proliferation, resulting in less tissue accumulation. Benefits associated with type 2 diabetes mellitus lie in better glucose tolerance, which reduces insulin resistance. Polyphenols reduce plasma glucose levels and, therefore, insulin secretion. Regarding dyslipidemia, in addition to decreasing circulating triglyceride levels, they also decrease the oxidation of LDL-cholesterol. This oxidation produces an imbalance in the levels of lipoproteins that lead to MetS, which is why its reduction is important. On the other hand, polyphenols are also involved in the regulation of inflammatory response and in the expression of genes involved with atherosclerosis, reducing the expression of these genes. They counteract the signaling of the cascade that forms atherogenic plaque, which is also beneficial for cardiovascular disease. It is through these mechanisms that a Mediterranean diet manages to reduce the main complications of MetS [37].

The dietary approaches to stop hypertension (DASH) are based on the consumption of whole grains, fruits and vegetables, and foods low in fat, especially saturated fatty acids and cholesterol. DASH has beneficial effects on NAFLD parameters such as triglyceridemia, insulin markers, and liver enzymes. It was recommended that participants maintain their physical activity routines, but no guidelines were given and no physical activity intervention was undertaken [38]. The current systematic review described a study [26] that recommended a Mediterranean dietary pattern to participants, based on foods such as fruit, vegetables, whole grains, legumes, fish, and other foods poor in saturated fatty acids. It demonstrates the improvement of NAFLD after a Mediterranean diet is followed, although it also recommended performing physical activity. In this case, although the diets have different names, it is obvious that they have a very similar basis in food consumption and nutrient contents. Hence, similar results were obtained, supporting the importance of diet to improve NAFLD.

As noted previously, people with a high probability of developing NAFLD are those with obesity and MetS, so all benefits that a Mediterranean diet achieves over MetS are directly related to those over NAFLD [16].

A Mediterranean diet, thanks to its compounds, reduces weight, body mass index, waist circumference, fasting insulin levels, homeostasis model assessment-insulin resistance, fatty liver indexes, triacylglycerides, fasting plasma glucose, and serum alanine aminotransferase. A few studies showed trends to decrease AST levels. Therefore, the beneficial effects of a Mediterranean diet could explain the improvement of NAFLD [39]. Moreover, it has been shown that there is a direct association between reduced insulin resistance and less severity in NAFLD [40]. All of these benefits are also evident in the articles included in this review, showing that compliance with a Mediterranean diet is associated with a lower probability of having NAFLD [21,22,24,27,29,30,31].

Another positive effect of a Mediterranean diet is that it has been shown to reduce intrahepatic lipids. The monounsaturated fatty acids of a Mediterranean diet, present for example in olive oil, improve lipid profile, insulin sensitivity, glycemic control, and blood pressure, producing an improvement in NAFLD. [40]. In fact, our findings show this relationship between a Mediterranean diet, the above benefits, and the improvement in NAFLD [22,26,29,30,31].

### 4.2. Mediterranean Diet, Physical Activity and NAFLD

Many studies showed the importance of regular physical activity for better health. A physical activity intervention can improve and normalize obesity, lipid disorders (lowering triglyceridemia and raising HDL-cholesterol), hypertension, and other MetS-related parameters. One of the major effects is on the normalization of insulin resistance. However, a physical activity intervention alone is not enough; it demands other elements such as cardiorespiratory fitness and an adequate diet. Cardiorespiratory fitness is the maximum ability of systems involved in exercise (mainly respiratory and cardiovascular) to supply oxygen to the skeletal muscles, and then to improve the activity. Thus, increasing regular physical activity and stimulating cardiorespiratory fitness may have a greater impact on health. It has been seen that an intervention of 3 to 12 months of exercise can improve levels of cardiorespiratory fitness and, consequently, reduce cardiovascular risk [16]. Although there was a possibility that these benefits were useful in slowing the progression of NAFLD, no specify details were given [16,21,27]. Following a Mediterranean diet improves MetS parameters and is directly associated with improved NAFLD [21,27]. For this reason, physical activity and Mediterranean diet go hand in hand.

Other literature highlighted the importance of physical activity and resistance training in achieving benefits with NAFLD. Physical activity provides benefits with NAFLD because it improves metabolic condition; however, the type and intensity of physical activity needed to achieve this effect is unclear [39]. The need for dietary intervention has been mentioned, as well as the importance of adding antioxidants into a Mediterranean diet to achieve beneficial effects with NAFLD [40]. However, it remains unclear which type of diet is the most appropriate in that case [19]. It is important to realize that not just any diet achieves beneficial effects and it is necessary to determine which type of diet is best. The current review shows that a Mediterranean diet is the most appropriate to decrease all risk factors for NAFLD and hence to improve this illness, since it is already rich in antioxidants and other healthy nutrients. However, the authors agreed that the beneficial effects of a Mediterranean diet are greater when dietary intervention is combined with the practice of regular physical activity [22,24,25,26,29,30,31,32,33]. Several studies showed that the effects of Mediterranean lifestyle would be obvious after one year of follow-up. It has also been reported that the intensity of physical activity is adaptable to each person’s physical condition and needs, in order to have a greater effect [26]. The ideal exercise would be an expenditure of 400 kcal for a person who weighs 70 kg for three days a week [26].

Some authors have described obesity as an independent risk factor for NAFLD. Weight, BMI, and, therefore, obesity, are directly related to a higher probability of developing NAFLD [41]. Our results show that this is not always sufficient, and that one can be obese without NAFLD, or vice versa [21,22]. The present review shows that the possibility of developing NAFLD increases with the expression of three or more MetS alterations. As mentioned, one of the MetS alterations is being overweight or obese, but it also includes others such as low HDL-cholesterol, hypertriglyceridemia, and high fasting glycemia, which further increase the possibility of the development and/or progression of NAFLD [21,22,23,24,25,26,27,28,29,30,31,32,33]. 

Recent literature supports lifestyle change to treat NAFLD, including dietary intervention and physical activity, but also bariatric surgery if necessary [42]. The current review shows that it is not necessary to put patients through the stress of surgery, since a properly applied Mediterranean diet is able to decrease weight and body mass index [24,29,30]. In fact, one of the consequences of any type of stress, including surgery, is an increase in oxidative stress with the production of reactive oxygen spices (ROS). They damage fatty acids and induce the lipid peroxidation chain reaction, which favors the development of NAFLD. This effect is counterproductive to the benefits sought with a Mediterranean lifestyle that seeks to reduce said stress with the contribution of antioxidants in a Mediterranean diet [43].

NAFLD not only affects adults, but there are also children with this pathology [44]. There is no clear therapy for treatment and pediatricians rely on dietary modification and/or physical activity [45]. They have studied several diets, all low in fat, but none based on a Mediterranean diet. They argued that there is insufficient evidence to recommend a single weight-loss method, although such weight loss improves NAFLD parameters. Accordingly, the current review supports the lack of therapies for NAFLD. However, it also supports the evidence that an effective treatment to improve NAFLD is to follow a Mediterranean diet together with physical activity, as part of a Mediterranean lifestyle. Both a Mediterranean diet and physical activity are considered equally important for the management of NAFLD.

A recent study, carried out in times of a pandemic, confirms the evidence from this systematic review. A large group of obese people, and a smaller group with diagnosed NAFLD, were interviewed about their lifestyle and anthropometric measurements during a month of confinement by COVID-19. It was found that weight had increased in more than half of the study subjects, who confessed that they did not engage in physical activity and had a low adherence to a Mediterranean diet. Furthermore, 70% of the NAFLD group also gained significant weight and thus did not receive any improvement in NAFLD. This confirms that situations where a Mediterranean lifestyle is not followed is a risk factor for metabolic diseases. At the same time, it confirms our results that both physical activity and Mediterranean diet are equally important in acting as a protective factor against this type of disease, including NAFLD [46].

## 5. Strengths and Limitations of the Study

The main strength of this review is that it provides up-to-date information about the association between NAFLD and a Mediterranean lifestyle. This review considers all aspects of lifestyle and treats them equally, including both physical activity and a Mediterranean diet. It does not only delve into one of two; it gives the same importance to both. In addition, diet is well detailed and specified: Mediterranean diet. Another strength is that it encompasses different types of study with different designs, which allows a more generalized and reliable view of the topic under different points of view. Some of the studies analyzed had a small sample size, providing insufficient statistical weight. The integration of these studies with others of bigger sample size in this review increases the final statistical power. However, there are some limitations. The first limitation is that there are some studies that applied intervention and classified participants into study and control groups, while others analyzed participants as a whole and did not apply intervention. Some of the included studies that separated the population into groups did not clarify the method used. The heterogeneity of the studies in terms of the length of follow-up, participant characteristics, different time points of data collection, and outcome measures is also another limitation. A bias to consider in this review relates to publication. All analyzed studies have been published, while the unpublished ones have not been considered. There is also attrition bias because the loss of participants (due to abandonment, non-compliance with the protocol, etc.) has not been considered. Another limitation is that searched literature has only been retrieved from the MEDLINE database, so it is very probable that some studies have not been included in this review.

## 6. Conclusions

One of the best treatments available to improve MetS and consequently NAFLD is the Mediterranean lifestyle: following a Mediterranean diet and regular physical activity. This has been shown to contribute greatly to reduce risk factors and to prevent, and even reverse, NAFLD. The effect is due to the benefits of the Mediterranean lifestyle on obesity, type 2 diabetes mellitus, high triglyceridemia, hypertension, and, in general, on disorders clustered under the name of MetS. Thus, hepatic steatosis and the amount of fat in the liver are reduced. There are still few studies that analyze in depth the combined effect of a specific intervention based on Mediterranean diet and physical activity, so this type of study in people with NAFLD and other types of metabolic disorders should be explored.

## Figures and Tables

**Table 1:** Description of the reviewed studies: study design and participants.

Author, Year (Reference)	Study’s Aim/Aims	Study Characteristics	Methods	Results
Mediterranean Diet and NAFLD				
Kim et al., 2016 [21]	Compare the components and prevalence of MetS according to degree of adiposity and presence of NAFLD	*n* = 1695 with a history of liver cirrhosis (70.5% female) Age 49–57 Korea Cross-sectional study	Collect and compare anthropometric, clinical, and laboratory data of non-obese males/females without NAFLD; non-obese males/females with NAFLD; obese males/females without NAFLD; and obese males/females with NAFLD	− ↑ Fasting glucose in non-obese participants with NAFLD vs. obese participants without NAFLD − ↑ 3.63 times prevalence of MetS with presence of NAFLD vs. ↑ 3.84 times prevalence of MetS with obesity without NAFLD (in males) − ↑ 5.56 times prevalence of MetS with presence of NAFLD vs. ↑ 3.46 times prevalence of MetS with obesity without NAFLD (in females)
Chen et al., 2019 [23]	Investigate the relationship between NAFLD risk and nut consumption	*n* = 1068 (534 with NAFLD and 534 without) (31.8% female) Age 18–70 China Retrospective case–control study	Collect dietary intake to calculate nut consumption. Categorize nut consumption in quartiles on the distribution of daily nut intake of controls	− No association between nut consumption and NAFLD risk in overall sample − Significant inverse association between ↑ nut consumption and NAFLD in the highest quartile of men’s sample
Georgoulis et al., 2015 [27]	Assess the presence of MetS and its association with dietary habits in subjects with NAFLD	*n* = 73 with NAFLD (31.5% female) Age 34–56 Athens Cross-sectional study	NAFLD diagnosed by high liver enzyme levels and ultrasound. Subjects’ food consumption assessed by food frequency questionnaire. Adherence to Mediterranean diet assessed by MedDiet Score	− 46.5% sample with MetS, ↑ waist circumference, and ↓ HDL − Positive association between MetS and consumption of red meat and refined grains − Negative association between MetS and MedDietScore and consumption of whole grains
Aller et al., 2018 [28]	Compare dietary, genetic, and biochemical parameters among obese and overweight participants with NAFLD	*n* = 203 with biopsy-proven NAFLD (43.3% female) Age 44–49 Spain Cross-sectional study	Evaluate adherence to Mediterranean diet using MEDAS questionnaire, anthropometrical and biochemical parameters, and the variants rs180069 of tumor necrosis factor gene and I148M of PNPLA3 gene	− ↑ Serum adiponectin levels and ↓ resistin and leptin concentration in overweight participants vs. obese participants − ↑ Frequency of NASH in obese participants − Adherence to Mediterranean diet as an independent protective factor for liver fibrosis and NASH in overweight participants
**Mediterranean Diet, Physical Activity and NAFLD**				
Konerman et al., 2018 [22]	Analyze the prevalence of NAFLD between subjects in the University of Michigan Metabolic Fitness (MetFit) Program and assess its impact on liver-related and metabolic parameters, and weight among subjects without and with NAFLD	*n* = 403 who completed the MetFit program at the University of Michigan between 2008 and 2016 (37.5% female) Age 45–63 Michigan Cohort study	Collect laboratory and clinical data at enrolment and at 12 and 24 weeks of subjects with and without NAFLD (defined based on imaging, liver biopsy, or clinical diagnosis) who have to follow a Mediterranean diet and exercise sessions	Principal group were men with severe obesity and NAFLD − 30% ↓ weight ≥ 5% − 62% resolution of hypertriglyceridemia − 33% resolution of low HDL − 27% resolution of impaired fasting glucose − 43% normalization of alanine aminotransferase
Sorrentino et al., 2015 [24]	Observe if, in participants with less advanced stages of NAFLD, a moderate regimen of diet, exercise, and a mix of vitamin E and a new formulation of silymarin could offer clinical improvements	*n* = 78 with MetS and ultrasound confirmation of liver steatosis (46.2% female) Age 55–57 Italy Controlled clinical study	90-days follow-up Group A: Standard Mediterranean diet, exercise, and a dietary adjunct (2 tablets/day of a nutraceutical product containing, in each tablet, 210 mg of Eurosil 85^®^) Group B: Standard Mediterranean diet and exercise.	Group A: ↓ BMI, abdominal circumference, ultrasound measurement of right liver lobe, HSI, and lipid accumulation product Group B: No change
Bullón-Vela et al., 2019 [25]	Examine the connection among NAFLD and lifestyle factors in participants with MetS	*n* = 328 with MetS who participate in PREDIMED-Plus study (45.1% female) Age 55–75 (men) and 60–75 (women) Spain Cross-sectional study	Collect dietary, clinical, and sociodemographic data. Evaluate physical activity and adherence to Mediterranean diet using validated questionnaires and NAFLD with non-invasive HSI	− ↓ HSI values with ↑ physical activity terciles − Adherence to Mediterranean diet inversely associated with HSI values − ↑ Terciles of legume consumption inversely associated with the highest tercile of HSI
Abbate et al., 2021 [26]	Examine the efficacy of lifestyle intervention on the reduction of MetS and NAFLD, and if these reductions could influence renal outcomes	*n* = 155 with MetS and NAFLD (39.1% female) Age 40–60 Spain Randomized controlled trial	6-months follow-up Group A (CD): Conventional diet based on American Association for the Study of Liver Disease recommendations with 10,000 steps a day Group B (MD-HMF): Mediterranean diet: high meal frequency (7 meals a day) with 10,000 steps a day Conventional Group C (MD-PA): Mediterranean diet: physical activity with instructed sessions 3 times a week	− No significant differences between 3 groups − ↓ Urinary albumin-to-creatine ratio in participants with ↑ levels at baseline, but without changes in liver fat − ↓ Estimated glomerular filtration in participants with hyperfiltration at baseline, associated with ↓ liver fat and insulin resistance and ↑ energy expenditure − Energy expenditure, ↓ hepatic fat accumulation, and insulin resistance = ↓ glomerular hyperfiltration − ↓ Increased albuminuria, without association with reduced liver fat
Gelli et al., 2017 [29]	Define the clinical effectiveness of nutritional recommendation on weight loss and the reduction of liver enzymes, anthropometric and metabolic indexes, and NAFLD	*n* = 46 with NAFLD (37% female) Age 26–71 Italy Observational study	Examine a Mediterranean diet and clinical intervention with physical activity over 6 months, monitoring and collecting metabolic parameters, liver enzymes, severity NAFLD (by ultrasound), cardiovascular risk indexes, and biochemistry at the middle of interventions and at the end	− ↓ 93% to 48% of percentage of participants with steatosis grade ≥ 2 − Regression of steatosis in 9 participants − 25 of 46 participants achieved a reduction of 7% of their weight or maintained a normal weight − ↓ Liver enzymes (especially alanine aminotransferase enzyme) − Improvement of waist circumference, BMI, waist-to-hip ratio, LDL/HDL, total cholesterol/HDL, triglycerides/HDL, serum glucose, HDL, fatty liver index, HOMA, Kotronen index, NAFLD liver fat score, visceral adipose index, and lipid accumulation product
Copaci et al., 2015 [30]	Examine if lifestyle intervention and exercise during a 12-month period could reduce weight and improve steatosis	*n* = 86 overweight with steatosis (40.7% female) Age 35–59 Romania Prospective observational study	12-months follow-up Caloric goal based on starting weight, daily fat goal, and physical activity (moderate intensity)	− ↓ Weight, BMI, waist circumference − ↓ Gamma glutamyl transferase, alanine aminotransferase, cholesterol, LDL, HOMA-R − Steatotest improved − Modification of leptin and adiponectin as factors related to improved steatosis (BMI and alanine aminotransferase also)
Takahashi et al., 2015 [31]	Examine the effects of resistance exercise on metabolic parameters of NAFLD	*n* = 53 with NAFLD (64.2% female) Age 37–68 Japan Randomized controlled study	12-months follow-up Group A: 12 weeks of resistance exercise and regimen Group B: Lifestyle counseling (dietary restrictions and regular physical activities)	Group A: ↑ Muscle mass and fat-free mass ↓ Mean insulin and ferritin levels, hepatic steatosis grade, HOMA-IR index Group B: ↓ LDL
Lee et al., 2018 [32]	Examine the association between NAFLD index and HGS in older adults	*n* = 538 with NAFLD (80.3% female) Age > 60 Korea Cross-sectional study	High HGS / Mid HGS / Low HGS groups (based on relative HGS) High risk / Low risk groups (based on FIB-4, SNS, HSI, and NFS) Assess body-composition parameters, HGS, and NAFLD	− ↓ Linear in NAFLD index (SNS, HSI, NFS, FIB-4) across ↑ HGS levels − Low HGS group: ↑ ORs of SNS, HSI, and NFS (compared to High HGS group)
Cho et al., 2021 [33]	Investigate the effect of HGS and SES on the risk of NAFLD in middle-aged adults	*n* = 5272 who participated in KNHANES (68.2% female) Age 53–61 Korea Cross-sectional study	NAFLD defined by HSI and comprehensive NAFLD score. SES based on self-reported questionnaire. Assessment of anthropometric data, blood markers, health-related factors, and HGS	↑ Risk of NAFLD in subjects with ↓ SES and HGS vs. subjects with ↑ SES and HGS

BMI = body mass index, FIB-4 = fibrosis 4 calculator, HDL = high-density lipoprotein, HGS = hand-grip strength, HOMA = homeostasis model assessment, HSI = Hepatic Steatosis Index, KNHANES = Korea National Health and Nutrition Examination Surveys, LDL = low-density lipoprotein, MEDAS = Mediterranean diet adherence screener, MedDietScore = Mediterranean diet score, MetS = metabolic syndrome, NAFLD = non-alcoholic fatty liver disease, NASH = non-alcoholic steatohepatitis, NFS = NAFLD fibrosis score, ORs = odds ratio, PNPLA3 = patatin-like phospholipase domain containing 3, SES = socioeconomic status, SNS = simple NAFLD score. ↑: Increase; ↓: Decrease.

## Data Availability

There are restrictions on the availability of data for this trial, due to the signed consent agreements around data sharing, which only allow access to external researchers for studies following the project purposes. Requestors wishing to access the trial data used in this study can make a request to pep.tur@uib.es.
